# Prediction of residual ischemic risk in ticagrelor-treated patients with acute coronary syndrome

**DOI:** 10.1186/s12959-022-00380-4

**Published:** 2022-04-21

**Authors:** Yuting Zou, Yuyan Wang, Yangxun Wu, Shizhao Zhang, Haiping Liu, Tong Yin

**Affiliations:** 1 Institute of Geriatrics, The Second Medical Center & National Clinical Research Center for Geriatric Diseases, Chinese PLA General Hospital, 100853 Beijing, China; 2 Medical School of Chinese PLA, Chinese PLA General Hospital, 100853 Beijing, China; 3 Department of Cardiology, the 6th Medical Center, Chinese PLA General Hospital, Beijing, China

**Keywords:** Ticagrelor, Platelet inhibition rate, Thromboelastography, Acute coronary syndrome

## Abstract

**Background:**

Despite strong antiplatelet therapy with ticagrelor, serious ischemic events still occur in patients with acute coronary syndrome (ACS). The predictability of platelet reactivity to the residual risk of ischemic events during ticagrelor treatment remains uncertain.

**Objectives:**

We aimed to investigate the predictability of the thromboelastography (TEG)-measured adenosine disphosphate (ADP)-induced platelet inhibition rate (ADP%) to the ischemic events in ticagrelor-treated patients with ACS.

**Methods:**

A cohort of ticagrelor-treated patients with ACS were consecutively recruited. ADP% was measured by TEG after 3 days of ticagrelor maintenance treatment. The primary ischemic event was defined as rehospitalization for unstable angina (UA) within 1 year, and the secondary ischemic event was a composite of the primary ischemic event plus all-cause death, nonfatal myocardial infarction (MI), stent thrombosis, stroke, and unplanned revascularization within 1 year.

**Results:**

A total of 751 eligible patients with ACS were included in the analysis, with 336 patients randomly assigned to the derivation group and 415 to the validation group. The overall rates of primary and secondary ischemic events were 14.51% (*n* = 109) and 16.91% (*n* = 127), respectively. Compared to the patients without ischemic events, those with ischemic events had a significantly lower ADP% both in the derivation group (for primary ischemic events: 66.05% vs. 92.80%, *p* < 0.001; for secondary ischemic events: 66.05% vs. 93.20%, p < 0.001) and in the validation group (for primary ischemic events: 66.40% vs. 89.20%, *p* < 0.001; for secondary ischemic events: 66.90% vs. 89.20%, *p* < 0.001). Receiver operating characteristic curve (ROC) analysis showed that an ADP% < 76% was the optimal cut-off value for predicting 1-year primary ischemic events, with an area under the curve (AUC) of 0.80 (95% CI: 0.72–0.86, *p* < 0.001) in the derivation group and 0.77 (95% CI: 0.69–0.85, *p* < 0.001) in the validation group. The multivariate Cox regression hazard analysis consistently identified an ADP% < 76% as an independent predictor of primary ischemic events in the derivation group (HR: 8.21, 95% CI: 4.82–13.99, *p* < 0.001) and in the validation group (HR: 6.34 95% CI: 3.32–12.11, *p <* 0.001). There was also a strong association between an ADP% < 76 and the occurrence of secondary ischemic events in the derivation group (HR: 7.33, 95% CI: 4.47–12.00, *p <* 0.001) and in the validation group (HR: 4.76, 95% CI: 2.73–8.32, *p <* 0.001).

**Conclusion:**

The ADP-induced platelet inhibition rate measured by TEG could predict ischemic events in ticagrelor-treated patients with ACS.

## Introduction

Dual antiplatelet therapy (DAPT) with a P2Y12 receptor inhibitor combined with aspirin is the cornerstone for the prevention of thrombotic events in patients diagnosed with acute coronary syndrome (ACS). As a strong and reversible P2Y12 receptor inhibitor, ticagrelor provides faster, more consistent and stronger P2Y12 receptor inhibition [1]. According to the latest guidelines, it is reasonable to use ticagrelor in preference to clopidogrel for maintenance P2Y12 inhibitor therapy in patients with ACS [2, 3]. Despite the consistent effects and proven benefits of adding ticagrelor to aspirin therapy, a significant percentage of patients (8–17%) still experience ischemic events [4–6]. How to predict the occurrence of adverse events in ticagrelor-treated patients remains uncertain. Optimal antiplatelet therapy should involve an objective assessment of the individual thrombotic tendency based on the measurement of platelet reactivity [7]. Compelling evidence from observational studies confirmed the contribution of platelet function tests to the prediction of ischemic events in patients with ACS [8–12]. However, most of these studies were based on clopidogrel-treated patients, and the link between platelet reactivity and clinical events in ticagrelor-treated patients remains uncertain [13, 14]. In addition, platelet reactivity with ticagrelor, assessed by the vasodilator-stimulated phosphoprotein (VASP) index, was not found to be associated with bleeding or thrombotic events in ACS patients undergoing percutaneous coronary intervention (PCI) [13]. Therefore, whether platelet reactivity can be used as a surrogate endpoint for ticagrelor-treated patients remains uncertain. Indeed, apart from the inhibition of the P2Y12-ADP pathway, ticagrelor has an additional antiplatelet pathway via the increase in adenosine, which inhibits platelet reactivity via activation of the adenosine A_2A_ receptor [15, 16]. Therefore, the VASP index and VerifyNow P2Y12 assay, which assesses the inhibition of the P2Y12-ADP pathway, could not accurately reflect the overall antiplatelet effect of ticagrelor [17]. Thromboelastography (TEG) can measure the strength of a clot as a direct function of the maximum dynamic properties of fibrin-platelet binding via GP IIb/IIIa and the platelet contractile system. It can be used to globally assess platelet aggregation mediated by fibrinogen, and the contribution of platelet-fibrin interactions to thrombosis and haemorrhage [12]. Therefore, in the present study, we aimed to evaluate the relationship between the ADP-induced platelet inhibition rate (ADP%) assessed by TEG and ischemic events in ticagrelor-treated patients with ACS.

## Methods

### Patient recruitment

In this study, consecutive patients with ACS treated with ticagrelor and aspirin in the Department of Cardiology, the Chinese PLA General Hospital, from September 2013 to May 2017 were recruited. All patients were tested for antiplatelet reactivity represented by the TEG-measured ADP% value after 3 days of maintenance treatment with ticagrelor. Patients were excluded if they had contraindications for ticagrelor treatment, a history of cardiac arrest, a history of severe dyspnea, active bleeding, previous major bleeding history or recent intracranial haemorrhage, a platelet count less than 100 × 10^9^/L, or a recent serious infection. The protocol of the study was approved by the medical ethics committee of the Chinese PLA General Hospital, and the study was conducted in accordance with the Declaration of Helsinki. All included patients provided written informed consent.

### Platelet function measurement

The antiplatelet effect of aspirin and ticagrelor was assessed by using a Thromboelastography Hemostasis Analyzer (LEPU Medical, Beijing, China) with platelet mapping. Blood samples for TEG analysis were obtained 18–24 h after 3 days of maintenance treatment with ticagrelor. The blood samples were processed within 2 h after blood draw according to the standard operating procedure. Peripheral blood sample measurements were performed using the TEG system to assess the effects of antiplatelet therapy action via the arachidonic acid (AA) and adenosine diphosphate (ADP) pathways. The TEG system relied on the measurement of thrombin-induced clot strength to enable a quantitative analysis of platelet function. Blood was analyzed according to the manufacturer’s instructions. Then, 360 μL of neutralized blood was immediately added to a heparinase-coated tube and assayed in the TEG analyzer to measure the thrombin-induced clot strength (MA_Thrombin_), which was indicative of the baseline maximal platelet reactivity. Heparinized blood (340 μL) was added to a noncoated tube containing reptilase and activator F to generate a whole blood crosslinked clot in the absence of thrombin generation or platelet stimulation (MA_fibrin_). A third sample (340 μL) of heparinized blood was added to a nonheparinase-coated tube in the presence of activator F and ADP (2 μmol) to generate a whole blood-crosslinked clot with platelet activation (MA_ADP_). ADP-induced platelet inhibition was calculated with software according to the following formula: ADP-induced platelet inhibition rate (ADP%) = [1-(MA_ADP_–MA_fibrin_)/(MA _thrombin_– MA _fibrin_)] × 100% [18–20].

### Follow-up and endpoints

Patients were followed up by telephone at 1, 3, 6, and 12 months after discharge to identify the occurrence of adverse ischemic events. Primary ischemic events were defined as rehospitalization for unstable angina (UA) within 1 year. Secondary ischemic events were defined as a composite of all-cause death, nonfatal myocardial infarction (MI), stent thrombosis, nonfatal stroke, unplanned revascularization and rehospitalization for UA within 1 year. All-cause death was defined as death that occurred unexpectedly during the follow-up period as the result of an evident cardiac event, unexplained sudden death, or noncardiac cause [21]. Nonfatal myocardial infarction (MI; an increase in serum troponin I or in creatinine kinase-myocardial band (CK-MB) of at least twice the upper normal limits with at least 1 of the following: acute onset of prolonged [≥20 min] typical ischemic chest pain; ST-segment elevation of at least 1 mm in 2 or more contiguous electrocardiogram (ECG) leads, or ST-segment depression≥0.5 mm in ≥2 contiguous leads; or T-wave inversion>1 mm in leads with predominant R waves) [22]. Stent thrombosis was defined according to Academic Research Consortium criteria [21]. Nonfatal stroke was defined as focal loss of neurologic function caused by an ischemic or haemorrhagic event, with residual symptoms lasting at least 24 h [23]. Rehospitalization for UA was defined as 1) ischemic discomfort or equivalent symptoms requiring hospitalization within 48 h of symptoms, 2) a duration ≥10 min at rest, or in an accelerating pattern, 3) the presence of dynamic ST depression, ischemia on stress testing or significant epicardial coronary artery stenosis, and 4) a final diagnosis of myocardial ischemia [24].

### Statistical analysis

Of the 751 consecutively recruited patients who were eligible for the study, we randomly sampled 336 patients as the “derivation cohort” for the optimal cut-off value development of the ADP% value. The remaining 415 patients constituted the “validation cohort” used for testing the final cut-off value. Categorical variables are expressed as n (%), and continuous variables following a normal distribution are expressed as the mean ± SD, continuous variables not following a normal distribution are expressed as medians and interquartile ranges (IQRs). Categorical variables were compared using the χ^2^ test. The Kolmogorov–Smirnov test was used to check for the normal distribution of continuous data. Normally distributed continuous data were compared between groups using a t test. Nonnormally distributed continuous data were compared between groups using the Wilcoxon signed rank test. The association of the ADP% value with clinical outcomes was investigated with the Cox regression hazard model using univariate and stepwise multivariate analysis. A receiver operating characteristic (ROC) analysis was used to assess the ability of posttreatment platelet reactivity to distinguish between patients with and without ischemic event. The optimal cut-off value was calculated by determining the smallest distance between the ROC curve and the upper left corner of the graph. Survival analysis for patients with and without ischemic event according to the optimal cut-off level was performed using the Kaplan–Meier method, and the differences between groups were assessed by the log-rank test. All analyses were performed using SPSS (version 22.0, SPSS Inc., Chicago, IL) and R (version 3.4.3, http://www.r-project.org), and a 2-tailed *P* value < 0.05 was considered indicative of significance.

## Results

### Baseline characteristics

The baseline characteristics of the derivation and validation cohorts are depicted in Table 1. Compared with the patients in the validation group, those in the derivation group were much older (68.67 ± 6.61 vs. 58.28 ± 10.13) and had a higher prevalence of hypertension (65.48% vs. 58.07%). In the validation group, a higher percentage of patients were smokers (57.35% vs. 41.37%) and had prior MI (25.54% vs. 15.48%), and more patients were undergoing PCI (88.67% vs. 53.27%).

**Table 1:** Baseline characteristics of the study population

Characteristics	Derivation group (*n* = 336)	Validation group (*n* = 415)
Age, (mean ± SD) y	68.67 ± 6.61	58.28 ± 10.13
Male sex, n (%)	232 (69.05)	328 (79.04)
BMI, (mean ± SD) kg/m^2^	25.34 ± 3.33	25.94 ± 3.56
Cardiovascular risk factors		
Smoking, n (%)	139 (41.37)	238 (57.35)
Hypertension, n (%)	220 (65.48)	241 (58.07)
Hyperlipidemia, n (%)	103 (30.65)	121 (29.16)
Diabetes mellitus, n (%)	112 (33.33)	151 (36.39)
Other medical history		
Prior MI, n (%)	52 (15.48)	106 (25.54)
Prior PCI, n (%)	71 (21.13)	108 (26.02)
Prior CABG, n (%)	4 (1.19)	3 (0.72)
Arrhythmia, n (%)	24 (7.14)	17 (4.10)
Clinical diagnosis		
STEMI, n (%)	35 (10.42)	35 (8.43)
NSTEMI, n (%)	25 (7.44)	29 (6.99)
UA, n (%)	231 (68.75)	270 (65.06)
Undergoing PCI, n (%)	179 (53.27)	368(88.67)
Number of diseased vessels		
1-vessel disease, n (%)	44 (13.10)	129 (31.08)
2-vessel disease, n (%)	42 (12.50)	115 (27.71)
3-vessel disease, n (%)	92 (27.38)	80 (19.28)
Comorbidities		
Renal insufficiency, n (%)	14 (4.17)	10 (2.41)
History of stroke, n (%)	29 (8.63)	30 (7.23)
Laboratory examination		
LVEF, median (IQR) %	58.00 (52.00,63.00)	58.00 (52.00,63.00)
Creatinine, median (IQR) μ mol/L	79.55 (69.00,93.85)	78.80 (69.90,91.60)
Concomitant medication		
Tirofiban, n (%)	153 (45.54)	210 (50.60)
PPI, n (%)	183 (54.46)	228 (54.94)
Heparin, n (%)	161 (47.92)	172 (41.45)

*Abbreviations: BMI* Body mass index, *MI* Myocardial infarction, *PCI* Percutaneous coronary intervention, *CABG* Coronary artery bypass grafting, *NSTEMI* Non-ST-segment elevation myocardial infarction, *STEMI, ST*-segment elevation myocardial infarction, *UA* Unstable angina, *LVEF* Left ventricular ejection fraction, *PPI* Proton Pump Inhibitor

### The ADP-induced platelet inhibition rate measured by TEG

**Fig. 1:**
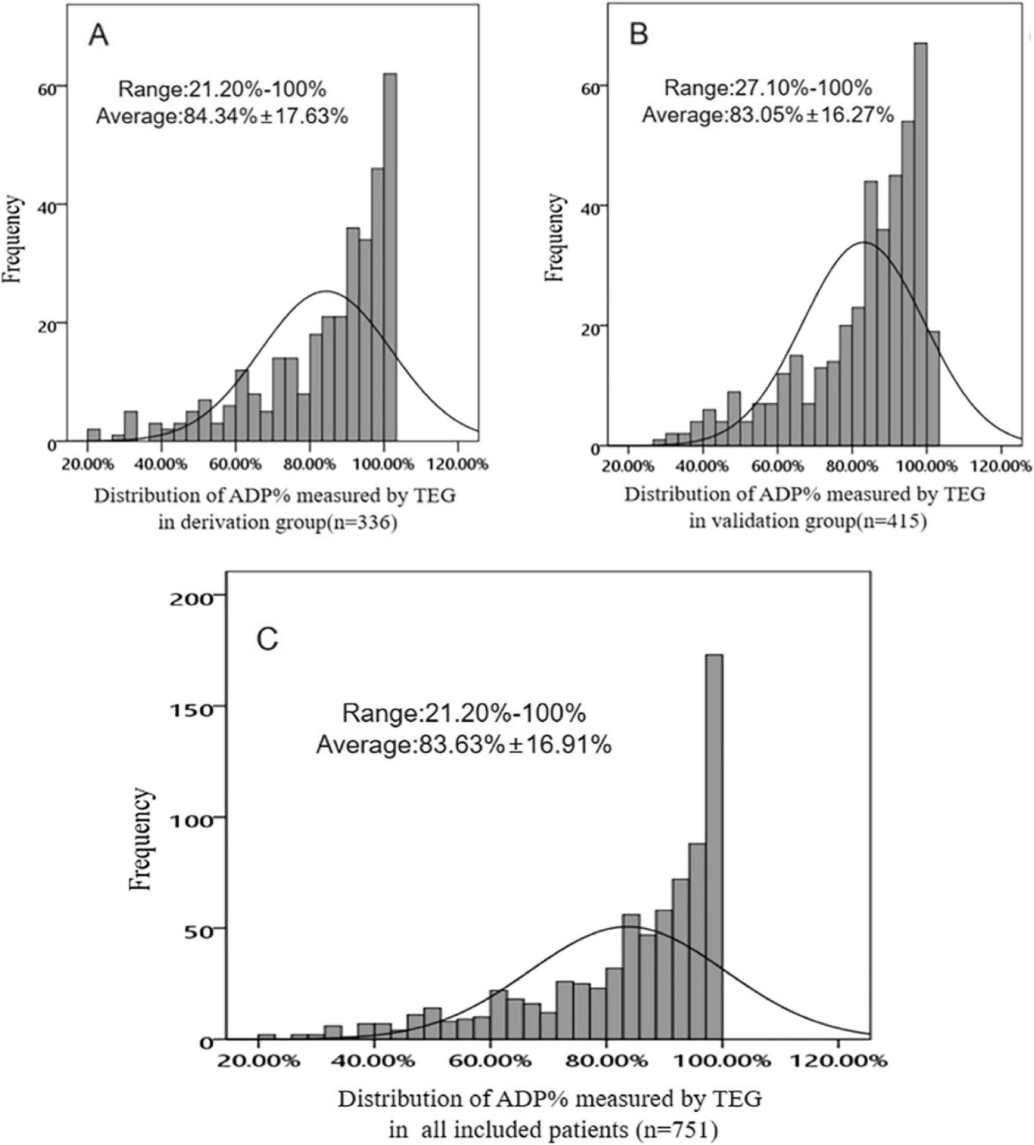
Distribution of ticagrelor platelet inhibiting rate measured by TEG in ACS patients. **A** and **B** represent the distribution of ADP% measured by TEG in derivation and validation group, respectively. **C** represent the distribution of ADP% in all included patients. ACS: acute coronary syndrome; ADP%: ADP-induced platelet inhibition rate; TEG: thromboelastography

After 3 days of maintenance treatment with ticagrelor, the distribution of ADP% values measured by TEG was skewed towards higher values (Fig. 1). The value of ADP% was 84.34% ± 17.63% on average (ranging from 21.20 to 100%) in the derivation group and 83.05% ± 16.27% on average (ranging from 27.10 to 100%) in the validation group (Fig. 1A, B). The value of the ADP% among all included patients was 83.63% ± 16.91% on average, ranging from 21.20 to 100% (Fig. 1C).

### Cut-off of the ADP-induced platelet inhibition rate for the prediction of ischemic events

**Fig. 2:**
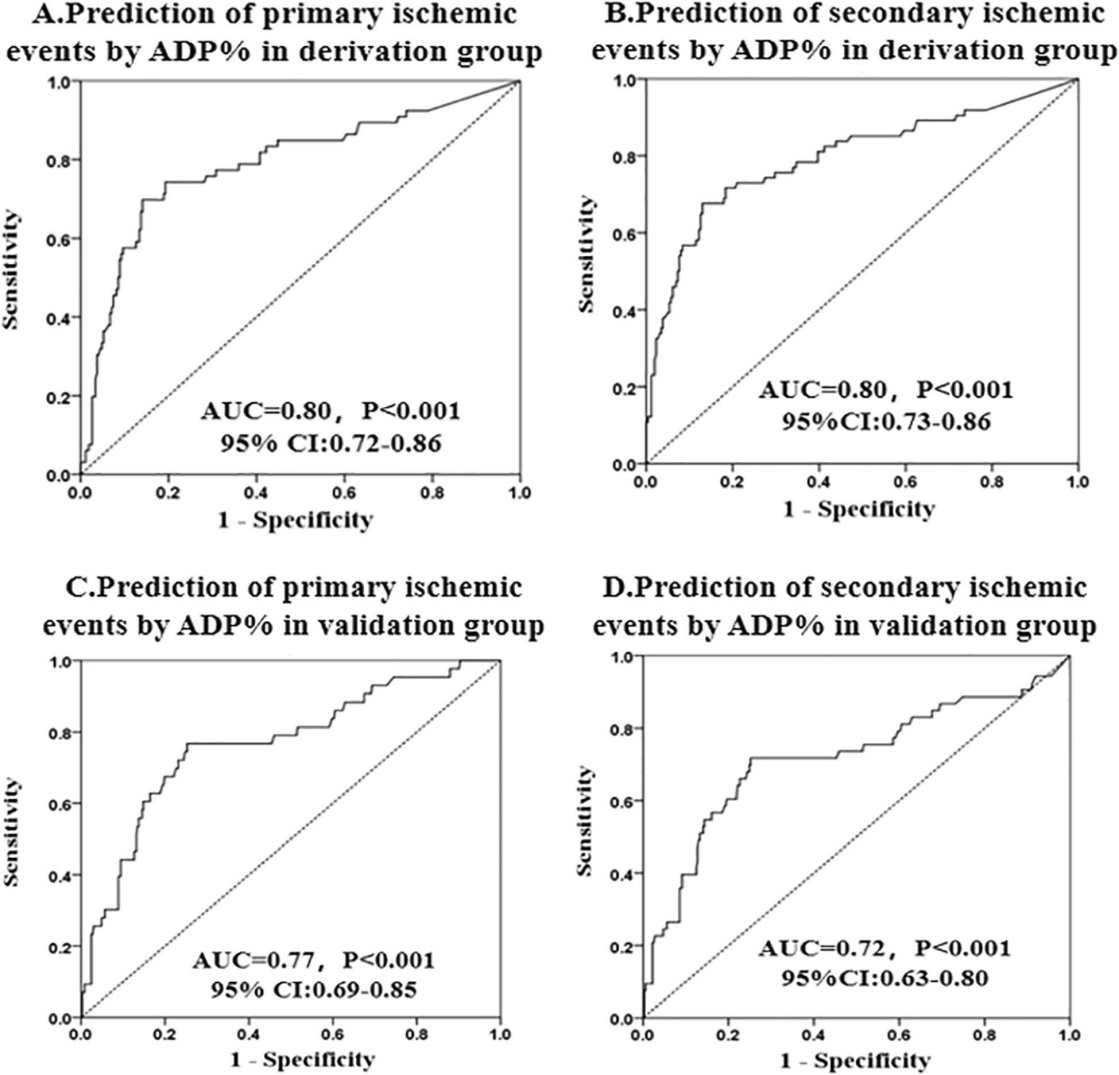
ROC curve for the prediction of ischemic events. A receiver-operating characteristic (ROC) analysis of ADP-induced platelet inhibition rate (ADP%) was used to distinguish patients with and without ischemic events. **A.** Prediction of primary ischemic events by ADP% in derivation group; **B**. Prediction of secondary ischemic events by ADP% in derivation group; **C.** Prediction of primary ischemic events by ADP% in validation group; **D.** Prediction of secondary ischemic events by ADP% in validation group. The primary ischemic events defined as the rehospitalization for unstable angina (UA) and the secondary ischemic events defined as the composite of rehospitalization for UA, all-cause death, nonfatal myocardial infarction (MI), stent thrombosis, stroke, and unplanned revascularization within 1 year. AUC: area under the curve

**Fig. 3:**
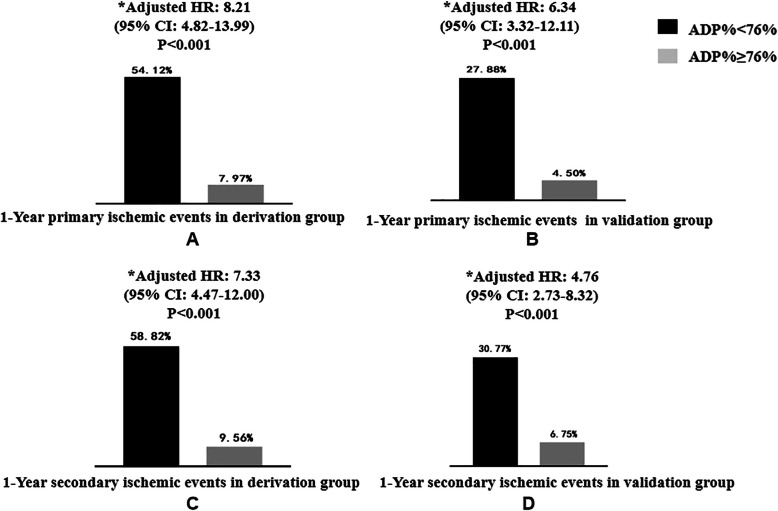
Clinical outcomes within 1-year follow-up in derivation and validation group. The ADP% independently correlated with both primary and secondary events by using the cutoff of < 76%. **A.** 1-Year primary ischemic events in derivation group; **B.** 1-Year primary ischemic events in validation group; **C.** 1-Year secondary ischemic events in derivation group; **D.** 1-Year secondary ischemic events in validation group. Primary ischemic events were defined as rehospitalization for unstable angina (UA). Secondary ischemic events were defined as a composite of rehospitalization for UA, all-cause death, nonfatal myocardial infarction (MI), stent thrombosis, nonfatal stroke, and unplanned revascularization. ^*^Adjusted by age, history of smoking, prior MI, hypertension, diabetes mellitus, and history of stroke

Within 1 year of follow-up, 66 (19.64%) primary ischemic events occurred in the derivation group, and 43 (10.36%) occurred in the validation group. Seventy-four (22.02%) secondary ischemic events occurred in the derivation group and 53 (12.77%) in the validation group. ROC analysis showed that TEG could distinguish primary ischemic events within the 1-year follow-up both in the derivation group (AUC = 0.80, 95% CI: 0.72 to 0.86, *P <* 0.001) and in the validation group (AUC = 0.77, 0.69 to 0.85, *P <* 0.001). Moreover, ROC analysis showed that TEG could distinguish secondary ischemic events within the 1-year follow-up in both the derivation group (AUC = 0.80, 95% CI: 0.73 to 0.86, *P <* 0.001) and the validation group (AUC = 0.72, 0.63 to 0.80, *P <* 0.001) (Fig. 2). Overall, an ADP% < 76% was identified as the optimal cut-off to predict the occurrence of primary ischemic events, with similar distributions in the derivation group and validation group. Using the cut-off value with the best sensitivity and specificity (ADP% < 76%), the proportions of primary ischemic events with an ADP% < 76 and ≥ 76% at 1 year were 54.12 and 7.97% (*p <* 0.001) in the derivation group and 27.88 and 4.50% in the validation group (*p <* 0.001), respectively (Fig. 3A and B). Using the cut-off value (ADP% < 76%), the proportions of secondary ischemic events with ADP% < 76 and ≥ 76% at 1 year were 58.82 and 9.56% (*p <* 0.001) in the derivation group and 30.77 and 6.75% in the validation group (*p <* 0.001), respectively (Fig. 3C and D).

### Contribution of the ADP-induced platelet inhibition rate cut-off to the prediction of ischemic events

**Fig. 4:**
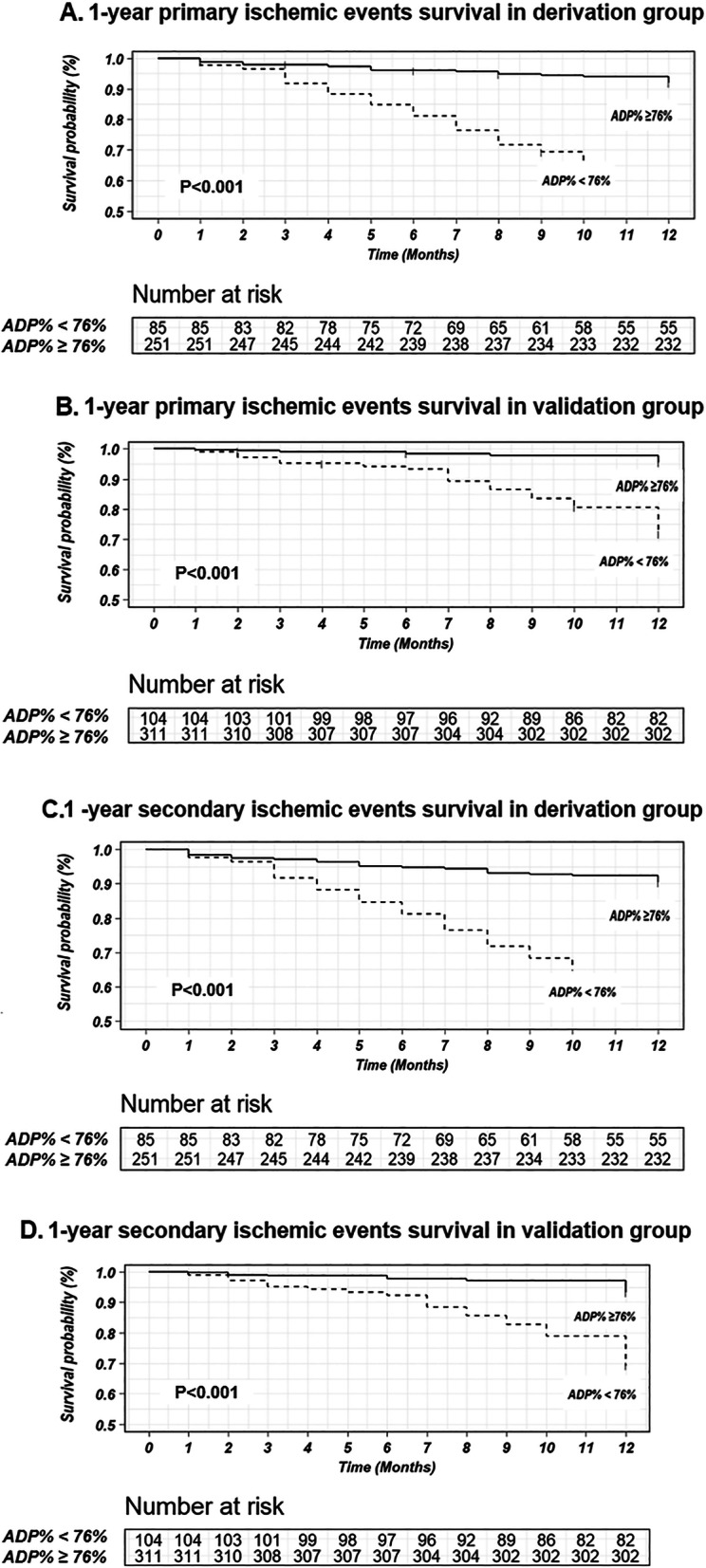
Survival free of ischemic events during 1-year follow-up in patients with ADP-induced platelet inhibition rate (ADP%) < 76% and with ADP% ≥76%. **A.** 1-year primary ischemic survival events survival in derivation group; **B.** 1-year primary ischemic events survival in validation group; **C.** 1-year secondary ischemic events survival in derivation group; **D.** 1-year secondary ischemic events survival in validation group. Primary ischemic events were defined as rehospitalization for unstable angina (UA). Secondary ischemic events were defined as a composite of rehospitalization for UA, all-cause death, nonfatal myocardial infarction (MI), stent thrombosis, nonfatal stroke, and unplanned revascularization

In multivariate Cox regression analysis, antiplatelet reactivity with ticagrelor, an ADP% < 76% measured by TEG, was associated with a higher risk of primary ischemic events in both the derivation group (adjusted HR: 8.21, 95% CI: 4.82–13.99; *p* < 0.001) and the validation group (adjusted HR, 6.34 95% CI, 3.32–12.11; *P* < 0.001) during the 1-year follow-up (Fig. 3A, B). An ADP% < 76% was also an independent risk factor for secondary ischemic events both in the derivation group (adjusted HR: 7.33, 95% CI: 4.47–12.00; *p <* 0.001) and in the validation group (adjusted HR, 4.76; 95% CI, 2.73–8.32; *P <* 0.001) (Fig. 3C, D). The event-free survival curves for the ischemic events according to an ADP% < 76% or ≥ 76% in the derivation group and validation group are shown in Fig. 4. One-year survival free from ischemic events was lower among patients with an ADP% < 76% in both the derivation group (*p <* 0.001) and the validation group (*p <* 0.001) (Fig. 4).

## Discussion

The main findings of the present study were that even with treatment with the more potent antiplatelet agent ticagrelor, antiplatelet variability as measured by TEG (ADP%) could be observed in patients with ACS. The cut-off value of ADP% < 76 could be used to predict the risk of residual ischemic cardiovascular events in ticagrelor-treated patients with ACS. To the best of our knowledge, this is the first study to establish the antiplatelet efficacy cut-off value of platelet reactivity for ticagrelor. The prediction of ischemic events with a platelet function test for ticagrelor-treated patients could facilitate the individualization of antithrombotic therapy.

Consistent with previous studies, the distribution of antiplatelet responsiveness measured after maintenance treatment with 90 mg ticagrelor twice a day was skewed towards higher values in the present study, indicating that the antiplatelet effect of ticagrelor was more potent and consistent than that of clopidogrel [25–28]. The evidence demonstrated that platelet reactivity with clopidogrel was correlated with clinical outcome in ACS patients. Therefore, a therapeutic window of platelet reactivity has been defined to predict both ischemic and bleeding events in clopidogrel-treated patients with ACS. However, according to the therapeutic window, no or less than 3.5% of ticagrelor-treated patients had high on-treatment platelet reactivity (HTPR) [27, 29]. Therefore, the hyporesponsiveness to ticagrelor leading to the high risk of ischemic events might be underestimated. According to the PLATO study, more than 9.8% of residual ischemic events (including myocardial infarction, stroke, or cardiovascular death) occurred in ticagrelor-treated patients with ACS [30], indicating that the current cut-off value of platelet reactivity based on clopidogrel is flawed for predicting the occurrence of ischemic events with ticagrelor treatment. It is necessary to establish a new cut-off value to assess the relationship between antiplatelet reactivity and the risk of ischemic events in ACS patients treated with ticagrelor.

Our study found that the risk of ticagrelor-related residual ischemic events could be predicted with the cut-off value of ADP% < 76 measured by TEG. The cut-off value in the present study had a strong discriminatory capability not only for rehospitalization for UA but also for secondary ischemic events (including all-cause death, nonfatal MI, stent thrombosis, nonfatal stroke, and unplanned revascularization). The incidence of rehospitalization for UA (14.51%) in our study was consistent with the ischemic event rate for the ticagrelor group in previous studies (1.7–20.0%) [4–6, 31]. This suggests that investigations should focus more on the individualized assessment of ischemia rather than on bleeding events alone. The cut-off value of ADP% < 76 suggested a higher risk of ischemic events among ticagrelor-treated ACS patients. In this situation, physicians might consider increasing the antiplatelet dose or extending the treatment time of ticagrelor for the prevention of ischemic events. Moreover, the use of GP IIb/IIIa inhibitors in addition to ticagrelor for ACS patients could reduce the rate of recurrent ischemic events [32, 33].

### Study limitations

First, the present study was a single-center observational study, which may not be fully representative of all clinical practice. Further large-scale studies should be carried out to explore the utility of TEG to personalize antiplatelet therapy to reduce the recurrence of ischemic events. Second, external validation of the study is warranted to confirm the practicality and reliability of the optimal cut-off value for the prediction of ischemic events. Third, the risk of bleeding events among ticagrelor-treated ACS patients could not be predicted with the value of ADP%. Further studies are needed to identify a novel platelet function test to predict the risk of bleeding events among ticagrelor-treated patients.

## Conclusions

The platelet inhibition rate with a cut-off value of 76% measured by TEG could be used to predict residual ischemic events in ticagrelor-treated patients with ACS.

## Data Availability

The data used to support the findings of this study are available from the corresponding author (yintong301@163.com) upon request.

## Abbreviations

AA: Arachidonic acid
ACS: Acute coronary syndrome
ADP: Acute myocardial infarction
ADP%: ADP-induced platelet inhibition rate
AUC: Area under the curve
CK-MB: Creatinine kinase-myocardial band
DAPT: Dual antiplatelet therapy
ECG: Electrocardiogram
HTPR: High on-treatment platelet reactivity
IQRs: Interquartile ranges
MA: Maximum amplitude
MA_ADP_: ADP-induced platelet-fibrin clot strength
MA _fibrin_: Fibrin- induced clot strength
MA_Thrombin_: Thrombin-induced clot strength
MI: Myocardial infarction
PCI: Percutaneous coronary intervention
ROC: Receiver operating characteristic curve
TEG: Thromboelastography
UA: Unstable angina
VASP: Vasodilator-stimulated phosphoprotein

## References

[1] Akhtar T, Bandyopadhyay D, Ghosh RK, Aronow WS, Lavie CJ, Yadav N (2020). Advances in the pharmacogenomics of antiplatelet therapy. Am J Ther.

[2] Levine GN, Bates ER, Bittl JA, Brindis RG, Fihn SD, Fleisher LA, Granger CB, Lange RA, Mack MJ, Mauri L, Mehran R, Mukherjee D, Newby LK, O’Gara PT, Sabatine MS, Smith PK, Smith SC (2016). ACC/AHA guideline focused update on duration of dual antiplatelet therapy in patients with coronary artery disease: a report of the American College of Cardiology/American Heart Association task force on clinical practice guidelines. J Am Coll Cardiol.

[3] Neumann FJ, Sousa-Uva M, Ahlsson A, Alfonso F, Banning AP, Benedetto U, Byrne RA, Collet JP, Falk V, Head SJ (2018). ESC/EACTS Guidelines on myocardial revascularization. Eur Heart J.

[4] Turgeon RD, Koshman SL, Youngson E, Har B, Wilton SB, James MT, Graham MM (2020). Association of Ticagrelor vs Clopidogrel with major adverse coronary events in patients with acute coronary syndrome undergoing percutaneous coronary intervention. JAMA Intern Med.

[5] Larmore C, Effron MB, Molife C, DeKoven M, Zhu Y, Lu J, Karkare S, Lieu HD, Lee WC, Vetrovec GW (2016). Real-World Comparison of Prasugrel With Ticagrelor in Patients With Acute Coronary Syndrome Treated With Percutaneous Coronary Intervention in the United States. Catheter Cardiovasc Interv.

[6] Effron MB, Nair KV, Molife C, Keller SY, Page RL, Simeone JC, Murphy B, Nordstrom BL, Zhu Y, McCollam PL, Vetrovec GW (2018). One-year clinical effectiveness comparison of Prasugrel with Ticagrelor: results from a retrospective observational study using an integrated claims database. Am J Cardiovasc Drugs.

[7] Gurbel PA, Tantry US (2009). Selecting optimal antiplatelet therapy based on platelet function monitoring in patients with coronary artery disease. Curr Treat Options Cardiovasc Med.

[8] Gurbel PA, Bliden KP, Guyer K, Cho PW, Zaman KA, Kreutz RP, Bassi AK, Tantry US (2005). Platelet reactivity in patients and recurrent events post-stenting: results of the PREPARE POST-STENTING study. J Am Coll Cardiol.

[9] Aradi D, Komocsi A, Vorobcsuk A, Rideg O, Tokes-Fuzesi M, Magyarlaki T, Horvath IG, Serebruany VL (2010). Prognostic significance of high on-clopidogrel platelet reactivity after percutaneous coronary intervention: systematic review and meta-analysis. Am Heart J.

[10] Sibbing D, Aradi D, Jacobshagen C, Gross L, Trenk D, Geisler T, Orban M, Hadamitzky M, Merkely B, Kiss RG, Komócsi A, Dézsi CA, Holdt L, Felix SB, Parma R, Klopotowski M, Schwinger RHG, Rieber J, Huber K, Neumann FJ, Koltowski L, Mehilli J, Huczek Z, Massberg S, Parma R, Parma Z, Lesiak M, Komosa A, Huczek Z, Koltowski L, Kowara M, Rymuza B, Klopotowski M, Malek L, Aradi D, Veress G, Dézsi AD, Merkely B, Lux Á, Kiss RG, Papp J, Kovács A, Dézsi CA, Amer S, Ruzsa Z, Róna S, Komócsi A, Ili R, Ungi I, Nagy F, Zweiker R, Tóth-Gayor G, Huber K, Haller P, von Scheidt W, Blüthgen A, Neumann FJ, Trenk D, Leggewie S, Kreider-Stempfle HU, Remp T, Kara K, Mügge A, Wutzler A, Fichtlscherer S, Zeiher AM, Seeger F, Hinterseer M, König A, Lederle S, Jacobshagen C, Czepluch F, Maier L, Schillinger W, Sossalla S, Hummel A, Felix S, Karakas M, Sydow K, Rudolph T, Halbach M, Gori T, Münzel T, May A, Gerstenberg CM, Pilecky D, Rieber J, Deichstetter M, Sibbing D, Mehilli J, Gross L, Kääb S, Löw A, Orban M, Orban M, Sattler S, Deuschl S, Teupser D, Holdt L, Mudra H, Räder T, Schütz T, Vahldiek F, Divchev D, Ince H, Nienaber CA, Radunski H, Boekstegers P, Horstkotte J, Mueller R, Geisler T, Müller K, Schwinger R, Rasp O (2017). Guided de-escalation of antiplatelet treatment in patients with acute coronary syndrome undergoing percutaneous coronary intervention (TROPICAL-ACS): a randomised, open-label, multicentre trial. Lancet.

[11] Breet NJ, van Werkum JW, Bouman HJ, Kelder JC, Ruven HJ, Bal ET, Deneer VH, Harmsze AM, van der Heyden JA, Rensing BJ, Suttorp MJ, Hackeng CM, ten Berg J (2010). Comparison of platelet function tests in predicting clinical outcome in patients undergoing coronary stent implantation. JAMA.

[12] Gurbel PA, Bliden KP, Navickas IA, Mahla E, Dichiara J, Suarez TA, Antonino MJ, Tantry US, Cohen E (2010). Adenosine diphosphate-induced platelet-fibrin clot strength: a new thrombelastographic indicator of long-term poststenting ischemic events. Am Heart J.

[13] Laine M, Panagides V, Frere C, Cuisset T, Gouarne C, Jouve B, Lemesle G, Paganelli F, Alessi MC, Mancini J, Bonello L (2021). On-Ticagrelor platelet reactivity and clinical outcome in patients undergoing percutaneous coronary intervention for acute coronary syndrome. Thromb Haemost.

[14] Laine M, Panagides V, Frere C, Cuisset T, Gouarne C, Jouve B, Thuny F, Paganelli F, Alessi MC, Mancini J, Bonello L (2019). Platelet reactivity inhibition following ticagrelor loading dose in patients undergoing percutaneous coronary intervention for acute coronary syndrome. J Thromb Haemost.

[15] Patrono C, Morais J, Baigent C, Collet JP, Fitzgerald D, Halvorsen S, Rocca B, Siegbahn A, Storey RF, Vilahur G (2017). Antiplatelet agents for the treatment and prevention of coronary Atherothrombosis. J Am Coll Cardiol.

[16] Gurbel PA, Bliden KP, Butler K, Tantry US, Gesheff T, Wei C, Teng R, Antonino MJ, Patil SB, Karunakaran A, Kereiakes DJ, Parris C, Purdy D, Wilson V, Ledley GS, Storey RF (2009). Randomized double-blind assessment of the ONSET and OFFSET of the antiplatelet effects of ticagrelor versus clopidogrel in patients with stable coronary artery disease: the ONSET/OFFSET study. Circulation.

[17] Grover SP, Bergmeier W, Mackman N (2018). Platelet signaling pathways and new inhibitors. Arterioscler Thromb Vasc Biol.

[18] Wu HY, Zhang C, Zhao X, Qian JY, Wang QB, Ge JB (2020). Residual platelet reactivity is preferred over platelet inhibition rate in monitoring antiplatelet efficacy: insights using thrombelastography. Acta Pharmacol Sin.

[19] Tang YD, Wang W, Yang M, Zhang K, Chen J, Qiao S, Yan H, Wu Y, Huang X, Xu B, Gao R, Yang Y, Yuan X, Ji H, Zhou Z, Liu Z, Chen J, Yuan J, Liu H, Qian J, Hu F, Shao C, Zhao H, Hua Y, Lu J (2018). Randomized comparisons of double-dose Clopidogrel or adjunctive Cilostazol versus standard dual antiplatelet in patients with high posttreatment platelet reactivity: results of the CREATIVE trial. Circulation.

[20] Zhao X, Li Q, Tu C, Zeng Y, Ye Y (2020). High glycated albumin is an independent predictor of low response to clopidogrel in ACS patients: a cross-sectional study. Cardiovasc Diabetol.

[21] Cutlip DE, Windecker S, Mehran R, Boam A, Cohen DJ, van Es GA, Steg PG, Morel MA, Mauri L, Vranckx P (2007). Clinical end points in coronary stent trials: a case for standardized definitions. Circulation.

[22] Cannon CP, Brindis RG, Chaitman BR, Cohen DJ, Cross JT, Drozda JP, Fesmire FM, Fintel DJ, Fonarow GC, Fox KA (2013). 2013 ACCF/AHA key data elements and definitions for measuring the clinical management and outcomes of patients with acute coronary syndromes and coronary artery disease: a report of the American College of Cardiology Foundation/American Heart Association Task Force on Clinical Data Standards (Writing Committee to Develop Acute Coronary Syndromes and Coronary Artery Disease Clinical Data Standards). Crit Pathw Cardiol.

[23] Coupland AP, Thapar A, Qureshi MI, Jenkins H, Davies AH (2017). The definition of stroke. J R Soc Med.

[24] Douglas PS, Hoffmann U, Patel MR, Mark DB, Al-Khalidi HR, Cavanaugh B, Cole J, Dolor RJ, Fordyce CB, Huang M (2015). Outcomes of anatomical versus functional testing for coronary artery disease. N Engl J Med.

[25] Alexopoulos D, Xanthopoulou I, Stavrou K, Hahalis G, Davlouros P (1743). Platelet reactivity measurements reveal patient noncompliance during ticagrelor maintenance therapy. Can J Cardiol.

[26] Alexopoulos D, Xanthopoulou I, Siapika A, Tsoni E, Stavrou K, Theodoropoulos KC, Davlouros P (2013). Evolving pattern of on-prasugrel and on-ticagrelor platelet reactivity over time in ST elevation myocardial infarction patients. Int J Cardiol.

[27] Alexopoulos D, Xanthopoulou I, Storey RF, Bliden KP, Tantry US, Angiolillo DJ, Gurbel PA (2014). Platelet reactivity during ticagrelor maintenance therapy: a patient-level data meta-analysis. Am Heart J.

[28] Yang Y, Chen W, Pan Y, Yan H, Meng X, Liu L, Wang Y, Wang Y (2020). Effect of ticagrelor versus clopidogrel on platelet reactivity measured by thrombelastography in patients with minor stroke or TIA. Aging (Albany NY).

[29] Laine M, Toesca R, Berbis J, Frere C, Barnay P, Pansieri M, Peyre JP, Michelet P, Bessereau J, Camilleri E, Helaf O, Camaleonte M, Paganelli F, Dignat-George F, Bonello L (2013). Platelet reactivity evaluated with the VASP assay following ticagrelor loading dose in acute coronary syndrome patients undergoing percutaneous coronary intervention. Thromb Res.

[30] Wallentin L, Becker RC, Budaj A, Cannon CP, Emanuelsson H, Held C, Horrow J, Husted S, James S, Katus H, Mahaffey KW, Scirica BM, Skene A, Steg PG, Storey RF, Harrington RA (2009). Ticagrelor versus clopidogrel in patients with acute coronary syndromes. N Engl J Med.

[31] Li XY, Su GH, Wang GX, Hu HY, Fan CJ (2018). Switching from ticagrelor to clopidogrel in patients with ST-segment elevation myocardial infarction undergoing successful percutaneous coronary intervention in real-world China: occurrences, reasons, and long-term clinical outcomes. Clin Cardiol.

[32] Cuisset T, Frere C, Quilici J, Morange PE, Mouret JP, Bali L, Moro PJ, Lambert M, Alessi MC, Bonnet JL (2008). Glycoprotein IIb/IIIa inhibitors improve outcome after coronary stenting in clopidogrel nonresponders: a prospective, randomized study. JACC Cardiovasc Interv.

[33] Karathanos A, Lin Y, Dannenberg L, Parco C, Schulze V, Brockmeyer M, Jung C, Heinen Y, Perings S, Zeymer U, Kelm M, Polzin A, Wolff G (2019). Routine glycoprotein IIb/IIIa inhibitor therapy in ST-segment elevation myocardial infarction: a Meta-analysis. Can J Cardiol.

